# A severe course in paediatric acute haematogenous osteomyelitis: Predictor variables present on admission

**DOI:** 10.1177/18632521251346650

**Published:** 2025-06-24

**Authors:** Marí Thiart, Pieter Nel, Jacques du Toit, Marilize Burger, Nando Ferreira

**Affiliations:** 1 Division of Orthopaedic Surgery, Department of Surgical Sciences, Faculty of Medicine and Health Sciences, Stellenbosch University, Tygerberg Campus, Cape Town, South Africa; 2 Division of Microbiology, Department of Pathology, Faculty of Medicine and Health Sciences, Stellenbosch University, Tygerberg Campus, Cape Town, South Africa

**Keywords:** Osteomyelitis, acute haematogenous, paediatric, predictor variables, severe clinical course

## Abstract

**Background:**

A subset of children with acute haematogenous osteomyelitis become severely ill. This study aimed to define a severe and standard course and identify potential risk factors on admission for a severe course as well as the cumulative incidence.

**Methods:**

This retrospective cohort study included all children under 16 years with acute haematogenous osteomyelitis between January 2018 and September 2021. The outcome parameters included >2 surgical debridements, C-reactive protein level not halving in 48 h, extraosseous involvement and hospital stay >14 days. Predictor variables (delayed presentation (>5 days), C-reactive protein >250 mg/L on admission, >1 bone segment and need for intensive care unit on admission) were tested against the outcome of a severe clinical course using univariate logistic regression analysis (using *p* < 0.2).

**Results:**

One hundred and twenty-one patients were included. Thirty-nine patients (32.2%) had a complicated course. Patients admitted to intensive care unit had a 2.8-times higher risk of a severe course compared to those not requiring intensive care unit (risk ratio 2.8; 95% confidence interval 1.6–4.8); having a C-reactive protein >250 mg/L on admission increased the risk of a severe course 1.7 times (risk ratio 1.71, 95% confidence interval 1.3–2.3). Having more than one bone segment involved and a delayed presentation of >5 days increased risk of a severe course by 2.4 (risk ratio 2.4, 95% confidence interval 1.6–3.6) and 1.3 times (risk ratio 1.3, 95% confidence interval 1.3–1.3)﻿, respectively, compared to the alternative. The cumulative incidence of acute haematogenous osteomyelitis ranged between 4.0% and 5.0% per year.

**Conclusion:**

Four risk factors present on admission were identified and are suggested to modify the risk of a severe disease as well as change treatment protocols.

## Introduction

Acute haematogenous osteomyelitis (AHOM) is a bacterial infection of bone or bone marrow and is predominantly a disease of childhood.^1^ Treatment of AHOM is multidisciplinary and includes orthopaedic surgeons, paediatricians and infectious diseases specialists.^2^ Interestingly, most AHOM infections occur in healthy children with no predisposing conditions.^3–6^

A subset of children with AHOM become severely ill and septicaemic, which could lead to pulmonary emboli, deep vein thrombosis (DVT) and multiorgan failure needing intensive care.^7–9^ It is important to identify this subset of children experiencing more severe symptoms early, to ensure that they receive prompt and effective management preventing complications and reducing morbidity. The ability to differentiate between mild and severe illness as early as possible also has the potential to impact resource utilisation.^10^

This ‘severe’ infection has not been clearly defined in the literature, and there is limited guidance for clinicians on how to pre-empt a more severe course of AHOM on admission. In a low- to middle-income country (LMIC), like South Africa, AHOM does not present as it does in most high-income countries. The patients present with a swollen limb containing an abscess, and some patients can be systemically ill with extraosseous involvement. The reason for the different presentation is multi-factorial and includes low socio-economic circumstances (parents might not have the finances to pay for transport to the hospital as the overburdened ambulance services cannot be relied upon) with far distances to travel.^11–13^ All patients require surgical debridement to drain the sub-periosteal (sometimes already spreading into the soft tissue) abscess. Those systemically ill are co-managed between paediatric infectious diseases and orthopaedic surgery.

The cumulative incidence of AHOM in our local setting is unknown. Knowing the cumulative incidence is important because it could change local funding allocation as well as raise awareness of AHOM in our community.

Woods et al. defined a complicated course as a patient having two or more bones involved, additional soft tissue sites, slow response to treatment, requiring multiple surgeries, bacteraemia for more than 3 days, the presence of a DVT and the presence of endocarditis.^14^ However, the author did not indicate how many of these variables are required to be considered a complicated course. An uncomplicated course was defined by the same team as a rapid resolution of bacteraemia, only one bone being involved, rapid (3–5 days) response to treatment and no acute sequelae.^14^ Reference was also made to the C-reactive protein (CRP) level declining after 48 h of treatment or a 50% decline from the peak CRP.^14^ Jagodzinski et al. reported that a CRP >100 mg/L was the best predictor for the need for prolonged intravenous antibiotics.^15^ This statement regarding CRP has since been widely accepted as a predictor for concomitant infections, prolonged intravenous antibiotics and increased risk for complications.^1,16–21^

This study aimed to define severe (complicated) and standard (uncomplicated) courses of disease as well as identify potential risk factors present on admission for a severe (complicated) course. A secondary objective was to report the cumulative incidence of AHOM in our LMIC setting.

## Materials and methods

This study followed a retrospective cohort design and considered all children who presented to a tertiary institution between January 2018 and September 2021. Institutional ethics approval was obtained before data collection (N21/07/067). All children under the age of 16 years, presenting with suspected AHOM, were included. Whilst the study setting is a tertiary referral centre in an LMIC, it is also the first contact for patients in the immediate geographical area. Referral of patients to the tertiary centre may happen for multiple reasons ranging from inadequate infrastructure at the local hospital (including lack of available theatre time, surgical or anaesthetic expertise or lack of special radiological investigations) to requiring admission to an intensive care unit (ICU). Children who presented with septic arthritis with no evidence of AHOM were excluded. AHOM in our setting rarely presents without a drainable abscess, and thus, no patient is admitted and treated with antibiotics alone. All patients, co-managed with paediatric infectious diseases specialists, were also included.

Hospital records, including electronic notes and radiographs, were accessed to identify eligible participants and were cross-referenced using the National Health Laboratory Service and Corporate Data Warehouse/Academic Affairs and Research Management System laboratory records. Demographic information was collected as well as specific clinical data points including the number of symptomatic days preceding the admission, presenting signs and symptoms, the bone segment(s) involved and the presence/absence of concomitant joint infection.

Management information that was collected included inpatient treatment such as the number of surgical debridements, the presence of septicaemia and extraosseous involvement (i.e. pneumonia, DVT, cardiac vegetations as identified on an echocardiogram). In addition, the choice and duration of antibiotics and whether the patient required intensive care was recorded.

All laboratory marker information was collected and included blood and tissue or fluid specimens sent for microscopy, culture and sensitivity analysis. The antibiograms were recorded for all positive cultures.

A severe (complicated) course was defined using clinical parameters based on the existing literature,^9–10,14,22–24^ clinical expertise, patient characteristics and receiver operator curve analysis. As an outpatient parenteral antimicrobial therapy service is not available in our country, more than 14 days was chosen as a substitute for a prolonged duration of intravenous treatment.

A severe (complicated) clinical course was, therefore, defined as requiring three of the four outcome variables, adapted from the definition by Woods et al.^14^:

- Requiring more than two surgical debridements (initial debridement and one relook debridement would be considered standard treatment).
- A CRP that does not halve within 48 h after surgical debridement and initiation of empiric antibiotics.
- Extraosseous involvement, including pneumonia, DVT and the presence of cardiac vegetations, at any stage during the hospital admission.
- Prolonged hospital stay (>14 days).^25,26^

A standard (uncomplicated) clinical course was defined as:

- Requiring one initial debridement and one relook debridement (number of surgical debridements ≤2).
- CRP that halves within 48 h after surgical debridement and initiation of empiric antibiotics.
- No systemic involvement

Data were captured onto an Excel spreadsheet and analysed using STATISTICA v14 (TIBCO Software Inc.) and STATA v15 (StataCorp LLC) software. Continuous data were tested for normality and were reported using means ± standard deviations with 95% confidence intervals (CIs) or as medians with interquartile ranges, for parametric and non-parametric data respectively. Categorical variables were described using counts and frequencies.

Cumulative incidence was calculated as AHOM divided by all paediatric admissions presenting to orthopaedics and admitted to our tertiary hospital over the study period.

The sample size to investigate the risk factors was determined by utilising the rule of thumb of 10 events per predictor variable considered. Approximately 11 predictor variables (age, time to presentation, CRP, erythrocyte sedimentation rate, white cell count, human immunodeficiency virus (HIV), presence of septicaemia, number of surgical debridements, systemic involvement, organism cultured and duration of hospital stay) were all considered to be important to predict the risk of a severe course. However, the available cohort size was anticipated to not be large enough to be sufficiently powered. Therefore, predictor variables of interest were reduced, based on clinical justification, into four final categorical predictor variables:

- Delayed presentation >5 days^27–29^
- A CRP on admission of >250 mg/L
- The need for ICU care at the time of admission due to the need for inotropes or respiratory support
- The number of bone segments involved: <2 versus ≥2^14^

Predictor variables were individually tested against the outcome of having a severe clinical course (three out of the four outcome variables) using univariate logistic regression analysis. A *p* < 0.2 was used to select variables to take forward into a multivariable model. Backwards and forwards stepwise selection methods were used to determine a final logistic regression model, which included only predictors with *p* < 0.05.

## Results

**Figure 1.**
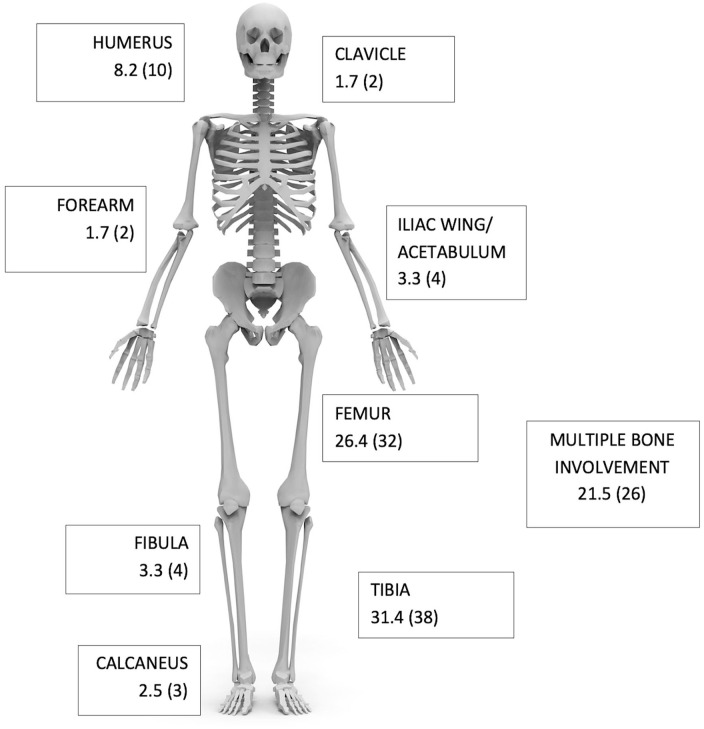
Schematic illustration of bone segments involved for all patients (*n* = 121). Data are presented as frequency and (count) for each bone segment.

A final cohort of 121 patients (90 males, 74.4%; 31 females, 25.6%) were included, and their demographic data are presented in Table 1. Fifty-five percent (*n* = 67 of 121) of patients were tested for HIV, of which 94.0% (*n* = 63 of 67) were negative. The tibia and femur were the most common bones involved (Figure 1). No patients in this cohort had spinal involvement. Whilst not all patients received a whole-body MRI to look for disseminated disease, none reported symptoms that would suggest vertebral or spinal cord involvement. All neurological symptoms reported resulted from cerebral involvement.

**Table 1.** Basic demographic data and presenting information of all patients presenting with AHOM (*n* = 121), those with a standard course (*n* = 82) and those with a severe course (*n* = 39).

Variable	All patients		Standard course		Severe course		*p* value^a^
	*N* = 121	Range	*N* = 82	Range	*N* = 39	Range	
Age (years)	7.0 (4.0–10.0)	0.0–15.0	7.0 (3.0–10.0)	0.0–14.0	8.0 (5.0–11.0)	0.0–15.0	0.045
Sex (male, %)	74.4 (90)	–	74.4 (61)	–	74.4 (29)	–	>0.999
Delay in presentation (days)	4.0 (3.0–7.0)	1.0–28.0	4.0 (2.5–7.0)	1.0–28.0	5.0 (3.0–7.0)	1.0–14.0	0.147
Admission WCC (×10^9^/L)	15.4 (11.3–20.2)	2.0–42.0	15.6 (11.1–20.1)	2.0–41.2	14.9 (9.5–20.5)	2.6–14.0	0.642
Admission CRP (mg/L)	229.0 (227.0–311.0)	22.0–502.0	200.5 (97.0–279.0)	22.0–502.0	289.0 (222.0–339.0)	52.0–500.0	0.001
ESR (mm/h)	70.0 (46.0–103.0)	2.0–141.0	65 (44.0–100.0)	5.0–140.0	83.5 (53.0–120.0)	2.0–141.0	0.076
Number of bone segments	1.0 (1.0–2.0)	1.0–5.0	1.0 (1.0–1.0)	1.0–2.0	4.0 (3.0–7.0)	1.0–5.0	<0.001

AHOM: acute haematogenous osteomyelitis; CRP: C-reactive protein; ESR: erythrocyte sedimentation rate; WCC: white cell count.

Data are described as median (interquartile range) or frequency (count), with the range indicated.

a *p* value indicates differences between patients with a standard course versus those with a severe course of treatment.

Eight (*n* = 8 of 121; 6.6%) patients had concomitant infections (septic arthritis) of the nearest joint. A total of 39 (32.2%) patients were defined as having a severe course (i.e. three out of the four variables as defined by Woods et al.).^14^

Patients with a severe course were slightly older (*p* = 0.045), had a higher admission CRP (*p* = 0.001) and had more bone segments involved (*p* < 0.001) compared to those with a standard course (Table 1).

**Figure 2.**
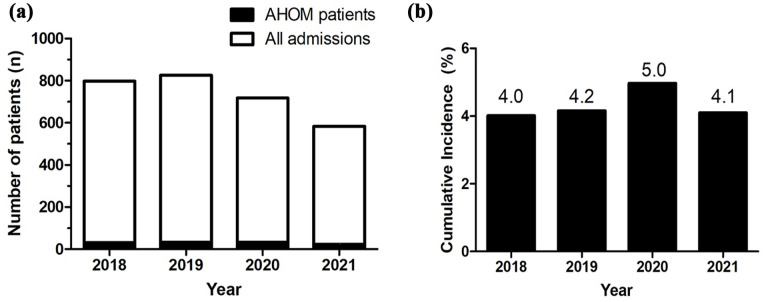
(a) The absolute number of admissions including those with AHOM. (b) The cumulative incidence of AHOM per study year. AHOM: acute haematogenous osteomyelitis.

The cumulative incidence of AHOM ranged between 4.0% and 5.0% per year, over the study period (Figure 2). These are only the patients admitted to our institution.

The most common organism cultured was *Staphylococcus aureus* – 63% of all blood cultures and 67% of all tissue samples. Ten patients (8.3%), of which seven experienced a severe course, had a polymicrobial sample, which included *S. aureus* (Table 2). Two patients had Gram-positive cocci seen on Gram stains but failed to culture an organism. The second most common organism was *Streptococcus pyogenes* isolated from five patients (4.1%). A total of 34 out of 39 (87.2%) patients who had a severe course cultured *S. aureus* (Table 3). Two patients cultured methicillin-resistant *S. aureus* (MRSA) but neither followed a severe course. As the focus of this study is not on the microbiology of AHOM patients, the antibiogram data are not shown.

**Table 2.** All specimens taken for microscopy, culture and sensitivity and the percentage of positive cultures.

Sample type	All patients			Standard course patients			Severe course patients		
	Sample sent (n)	Positive samples (%, *n*)	*S. aureus* positive (%, *n*)	Sample sent (*n*)	Positive samples (%, *n*)	*S. aureus* positive (%, *n*)	Sample sent (*n*)	Positive samples (%, *n*)	*S. aureus* positive (%, *n*)
Blood	92	74.0 (68)	68.5 (63)	55	61.8 (34)	54.5 (30)	39	87.2 (34)	79.5 (31)
Tissue	81	96.3 (78)	82.3 (67)	51	92.2 (47)	78.4 (40)	30	100.0 (30)	90.0 (27)
Fluid	21	95.2 (20)	76.2 (16)	14	92.9 (13)	71.4 (10)	7	85.7 (6)	85.7 (6)
Pus swab	19	94.7 (18)	73.7 (14)	17	100.0 (17)	92.9 (13)	2	50.0 (1)	50.0 (1)

*S. aureus: Staphylococcus aureus.*

Data are presented as counts or frequency (count).

**Table 3.** Analysis of inpatient data for all patients presenting with AHOM, between those with a standard course and those with a severe course.

Variable	All patients		Standard course		Severe course		*p* value^a^
	*N* = 121	Range	*N* = 82	Range	*N* = 39	Range	
Maximum CRP (mg/L)	276.0 (142.0–343.0)	27.0–502.0	220.0 (124.0–328.0)	27.0–502.0	322.0 (285.0–390.0)	52.0–500.0	<0.001
Percentage of CRP decrease after 48 h (%)	19.3 (−10.1 to 52.0)	−217.0 to 502.0	35.6 (−10.0 to 63.0)	−217.1 to 88.0	15.5 (−9.4 to 29.4)	−154.0 to 62.4	0.021
Number of surgeries	2.0 (2.0–4.0)	1.0–12.0	2.0 (1.0–3.0)	1.0–11.0	4.0 (3.0–7.0)	1.0–12.0	<0.001
Number of antibiotics	2.0 (2.0–3.0)	1.0–6.0	2.0 (1.0–3.0)	1.0–4.0	3.0 (2.0–3.0)	1.0–6.0	<0.001
Length of stay (days)	14.0 (9.0–21.0)	1.0−152.0	11.0 (8.0–14.0)	1.0–48.0	23.0 (18.0–39.0)	14.0–152.0	<0.001

AHOM: acute haematogenous osteomyelitis; CRP: C-reactive protein.

Data are described as median (interquartile range [IQR]) or frequency, or as a range. Counts are indicated in parentheses.

a *p* value indicates differences between patients with a standard course versus those with a severe course of treatment.

Nearly 40% of patients received two antibiotics (Table 4). The most common antibiotic used was cefazolin (62.0%) followed by cloxacillin (17.4%). The median length of stay was 14 days (interquartile range [IQR] 9–21) with a maximum of 152 days.

**Table 4.** In-patient and late complications observed in all patients with AHOM.

In-patient complications	*N* = 89 in-patient complications, *n*=50 patients	
	Standard course (% [*N*])	Severe course (% [*N*])
Septic shock	1.1 (1)	9.0 (8)
GIT^a^	2.2 (2)	5.6 (5)
Pulmonological^b^	2.2 (2)	20.2 (18)
Cardiovascular^c^	0.0 (0)	12.4 (11)
Anaemia requiring blood transfusion	7.9 (7)	14.6 (13)
Neurological^d^	1.1 (1)	5.6 (5)
Death	1.1 (1)	0.0 (0)
Acute kidney injury	1.1 (1)	1.1 (1)
Sickling crisis	0.0 (0)	1.1 (1)
Requiring skin coverage (split skin graft [SSG]/flap)	4.5 (4)	5.6 (5)
Additional imaging	3.4 (3)	0.0 (0)
Late complications	*N* = 29 late complications, *n* = 22 patients	
	Standard course (% [*N*])	Severe course (% [*N*])
Pathological fracture	17.2(5)	13.8 (4)
Deformity	10.3 (3)	3.4 (1)
Chronic osteomyelitis	13.8 (4)	3.4 (1)
Avascular necrosis of the femoral head	3.4 (1)	6.9 (2)
Hip dislocation from proximal femoral osteomyelitis	3.4 (1)	3.4 (1)
Readmission	13.8 (4)	6.9 (2)

AHOM: acute haematogenous osteomyelitis.

Data are presented as frequencies (counts).

a Gastrointestinal (GIT) complications included ileus and upper GIT bleeding.

b Pulmonological complications included pneumonia (*n* = 17), pleural effusion with associated pneumonia (*n* = 1) and pneumothorax (*n* = 2).

c Cardiovascular complications included constrictive pericarditis (*n* = 2) and deep vein thrombosis (*n* = 9).

d Neurological complications included meningitis (*n* = 4) and febrile convulsions (*n* = 1) and a peripheral nerve palsy (n = 1).

Of the patients who had a severe course, 61.5% (*n* = 24 of 39) were directly admitted to ICU on presentation. In the standard course cohort, only 14.6% (*n* = 12 of 82) were directly admitted to ICU on presentation.

There were significant differences between the maximum CRP (mg/L), the number of surgeries, the number of antibiotics as well as the length of stay between the two groups (Table 3). Interestingly, the percentage drop in CRP level between the groups was not statistically significant.

Fifty-five percent (*n* = 66) of patients developed complications (acute and chronic including any extra-osseous involvement). More than one complication was seen in 28 (23.2% of the total cohort) patients (the details of these are listed in Table 4). A total of 35 patients out of the 39 who had a severe course developed a complication (89.7%). Interestingly, none of the patients with a standard course developed a DVT. However, the only mortality occurred in the standard course group.

All independent risk factors were independently associated with an increased risk of a severe clinical course and therefore were carried forward into the multivariable regression analysis (Table 5). All independent risk factors remained in the final multivariable logistic regression model with an overinflated z-score (and thus a very narrow CI) for delayed presentation indicating the possible presence of effect modification between the predictor variables (Table 5).

**Table 5.** Bivariate and multivariable logistic regression analysis to investigate risk factors for a severe course of treatment.

Predictor variables present on admission	Standard course ((%) n)	Severe course ((%) n)	*p* value	Adjusted RR (95% CI)	*p* value
Delayed presentation					
≤5 days	57.5 (46)	44.4 (16)	0.192	1.3 (1.3–1.3)	<0.001
>5 days	42.5 (34)	55.6 (20)			
CRP at admission					
<250	69.5 (57)	33.3 (13)	<0.001	1.7 (1.3–2.3)	<0.001
>250	30.25 (25)	66.7 (26)			
Bone segments involved					
<2	91.5 (75)	53.8 (21)	<0.001	2.4 (1.6–3.6)	<0.001
≥2	8.5 (7)	46.2 (18)			
ICU on admission					
No	85.4 (70)	38.5 (15)	<0.001	2.8 (1.6–4.8)	<0.001
Yes	14.6 (12)	61.5 (24)			

CI: confidence interval; CRP: C-reactive protein; ICU: intensive care unit; RR: risk ratio.

Data are described as frequency (count).

## Discussion

The cumulative incidence of AHOM in this study ranged between 4% and 5%. Whilst cumulative incidence is not generally reported in the literature, crude comparisons can be drawn through the absolute number of patients that are reported on in various studies: in the present study, 121 children were treated over 45 months, which roughly equates to 32 children a year. Street et al. reported on 81 children treated in New Zealand per year,^30^ whilst Lindsay et al. reported on an average of 52 children per year in a study performed in Texas, USA.^31^ Popescu et al., in a study performed in Romania, reported on 94 patients treated over 10 years averaging nine patients per year.^32^ Whilst the number of patients in this cohort is lower than what has been reported in studies from New Zealand (813 patients in 10 years) and Texas, USA (209 patients in 4 years), it was higher than in European literature (94 patients in 10 years).^30–32^ It is, however, important to note that the population at risk is not considered when comparing absolute numbers, and this comparison should be interpreted with caution. In addition, it is also possible that this study’s reported cumulative incidence might be under-represented, due to regional hospitals being able to treat AHOM locally. As a result, the reported cumulative incidence reflects only those children residing in the direct geographic catchment area of the hospital, or those referred for tertiary (ICU) care.

Despite this study’s low incidence of AHOM, a third of the children in this study had a severe form of the disease, with all children included in the study requiring surgery. Complicated courses of AHOM as well as severity scores are mentioned in various studies.^2,10,24,33^ None of these studies, however, clearly define a ‘complicated course’ or ‘severe course’. Whilst Woods et al.^14^ suggested a definition in 2021, no indication was given of how many of the factors should be present to truly represent a severe course. In the present study, we adapted the suggested definition to include a measurable laboratory marker, CRP, halving within 48 h,^14^ as well as to propose meeting a minimum of three of the four criteria (Table 6).

**Table 6.** Comparison of the Woods et al. definition and the present study’s proposed definition of an uncomplicated and complicated course of treatment.

Characteristic	Woods et al.		The current study’s proposed definition	
	Uncomplicated	Complicated	Standard course	Severe course
Sites of infection	• Single bone	• Two or more bones involved • Additional soft tissue sites of infection beyond the bone	• Needing one initial debridement and one relook debridement • No systemic involvement	• More than two surgical debridements • Extraosseous involvement including pneumonia, deep vein thrombosis and cardiac vegetations
Clinical response to medical and surgical treatment	• Rapid (within 3–5 days)	• Slowed, prolonged response or lack of clinical response • Need for more than one surgery for source control	• A CRP that halves within 48 h after surgical debridement and initiation of empiric antibiotics	• A CRP that does not halve within 48 h after surgical debridement and initiation of empiric antibiotics
Course of bacteraemia when present	• Rapid resolution of bacteraemia (serial blood cultures become negative)	• Prolonged bacteraemia (3+ days)		
Acute sequelae of infection		• Venous thrombosis or septic thrombophlebitis • Endocarditis		• Prolonged hospital stay (>14 days)
Late sequelae of infection		• Findings concerning physeal injury • Pathological fracture		

CRP: C-reactive protein.

Four risk factors (delayed presentation >5 days; presence of raised CRP (>250 mg/L) on admission; ICU admission on presentation; ≥2 number of bone segments involved) were investigated for their potential role in predicting a more severe course of disease. It was interesting to note that delayed presentation as an exposure variable in the final model resulted in an overinflated *z*-score, and subsequently a very narrow CI. This finding might indicate the possibility of interactions between risk factor variables. Whilst statistically curious, this is clinically not surprising, given the complexity and difficulty of differentiating clearly between predictor variables and the definition of the outcome (i.e. a severe clinical course). Despite delayed presentation being commonly quoted in the literature as having a poorer outcome,^22,34^ having a delayed presentation only increased the risk of a severe course by 1.3 times compared to the patients who presented before 5 days of symptoms.

A lot of emphasis has been placed on the CRP levels in the literature. Arnold et al. have found an increase in CRP levels (median 174 mg/L) in patients with Methicillin-resistant Staphylococcus aureus (MRSA) infection when compared to CRP levels (118 mg/L) in patients with Methicillin-sensitive Staphylococcus aureus (MSSA) infection as well as Branson et al. (CRP 147 mg/ L on admission versus 88 mg/L).^35,36^ Concurrent AHOM and septic arthritis can lead to increased inflammatory markers, prolonged bacteraemia and hospital duration.^9,16^ In this study, neither MRSA nor concurrent infection seemed to be a significant marker of a severe course. The current study had only two patients with MRSA and neither had a severe course. Those with concurrent infections were relatively equally distributed with more in the standard course (five patients) than the severe course (three patients). A CRP on admission of ≥250 mg/L increased the risk for a severe course by 1.7 times, compared to the patients presenting with CRP levels on admission of <250 mg/L.

Athey et al. validated a severity of illness score in children with AHOM, but this score needs at least 4 days of data, and the authors stated that this score’s limitation was that it could not predict severity at the time of admission.^10^ They reported an increased score in children with MRSA, multifocal disease, more than two positive blood cultures, more than one surgical procedure, ICU admission, ICU care needed for more than 4 days and length of stay more than 21 days.^10^

In this study, the involvement of two or more bone segments increased the risk for a severe course by 2.4 times when compared to those with only one bone segment involved. The need for ICU admission based on cardiovascular instability or respiratory support indicates a severe disease of AHOM,^10,37^ and this is confirmed in the present investigation, where the need for ICU on admission increases the risk for a severe course to 2.8 times compared to those who did not require ICU care on admission.

The finding of more than two bone segments and the need for ICU care on admission increases the risk of a severe course more than delayed presentation and a CRP on admission >250 mg/L has not, to our knowledge, been reported previously.

Limitations of the current study are the small sample size, even if similar to previous investigations,^10^ and a small percentage of the clinical data was missing. The cumulative incidence can also be skewed as occasionally paediatric orthopaedic patients are admitted to other wards. Some patients with simple AHOM could have been treated in the peripheral hospitals if not requiring specialist or ICU care, which could also have skewed our cumulative incidence.

The unusual spike in the percentage of general admissions in 2020 could potentially be explained by the COVID outbreak where the paediatric orthopaedic ward was temporarily shared with general paediatrics who required admission for respiratory illnesses. We also have to note that no elective paediatric orthopaedic procedures were completed during the COVID-19 pandemic, and this could also skew our cumulative incidence result.

It should also be mentioned that we had a national shortage of cloxacillin, and thus, an increased proportion of patients were treated with cefazolin.

## Conclusion

A third of all the cases with AHOM had a severe course of disease. Four risk factors were identified and are suggested to, independently and collectively, modify the risk of a more severe disease progression. As all four of these risk factors are present on admission, this aids the clinician in identifying patients at high risk of severe disease, which can subsequently influence management decisions. This could include starting two synergistic antibiotics like cloxacillin and clindamycin, a whole-body MRI to look for other sources, an echocardiogram and duplex Doppler of the affected limbs and planning repeat surgical debridements.

## Supplemental Material

sj-pdf-1-cho-10.1177_18632521251346650 – Supplemental material for A severe course in paediatric acute haematogenous osteomyelitis: Predictor variables present on admission Supplemental material, sj-pdf-1-cho-10.1177_18632521251346650 for A severe course in paediatric acute haematogenous osteomyelitis: Predictor variables present on admission by Marí Thiart, Pieter Nel, Jacques du Toit, Marilize Burger and Nando Ferreira in Journal of Children’s Orthopaedics
